# Optical Characterization of Nano- and Microcrystals of EuPO_4_ Created by One-Step Synthesis of Antimony-Germanate-Silicate Glass Modified by P_2_O_5_

**DOI:** 10.3390/ma10091059

**Published:** 2017-09-09

**Authors:** Jacek Zmojda, Marcin Kochanowicz, Piotr Miluski, Agata Baranowska, Wojciech A. Pisarski, Joanna Pisarska, Renata Jadach, Maciej Sitarz, Dominik Dorosz

**Affiliations:** 1 Department of Power Engineering, Photonics and Lighting Technology, Bialystok University of Technology 45D Wiejska Street, 15-351 Bialystok, Poland; m.kochanowicz@pb.edu.pl (M.K.); p.miluski@pb.edu.pl (P.M.); gata0@onet.pl (A.B.); 2 Institute of Chemistry, University of Silesia, 9 Szkolna Street, 40-007 Katowice, Poland; wojciech.pisarski@us.edu.pl (W.A.P.); joanna.pisarska@us.edu.pl (J.P.); 3 Faculty of Materials Science and Ceramics, AGH University of Science and Technology, 30 Mickiewicza Av., 30-059 Krakow, Poland; rjadach@agh.edu.pl (R.J.); msitarz@agh.edu.pl (M.S.); ddorosz@agh.edu.pl (D.D.)

**Keywords:** glass-ceramics, europium oxide, luminescence properties, Stark splitting, EuPO_4_ nanocrystals, antimony-germanate-silicate glass

## Abstract

Technology of active glass-ceramics (GC) is an important part of luminescent materials engineering. The classic method to obtain GC is based on annealing of parent glass in proper temperature and different time periods. Generally, only the bulk materials are investigated as a starting host for further applications. However, the effect of an additional heat-treatment process on emission and structural properties during GC processing is omitted. Here, we focus on the possibility of obtaining transparent glass-ceramic doped with europium ions directly with a melt-quenching method. The influence of phosphate concentration (up to 10 mol %) on the inversion symmetry of local environment of Eu^3+^ ions in antimony-germanate-silicate (SGS) glass has been investigated. The Stark splitting of luminescence spectra and the local asymmetry ratio estimated by relation of (^5^D_0_→^7^F_2_)/(^5^D_0_→^7^F_1_) transitions in fabricated glass confirms higher local symmetry around Eu^3+^ ions. Based on XRD and SEM/EDX measurements, the EuPO_4_ nano- and microcrystals with monoclinic geometry were determined. Therefore, in our experiment, we confirmed possibility of one-step approach to fabricate crystalline structures (glass-ceramic) in Eu–doped SGS glass without additional annealing process.

## 1. Introduction

In photonic material technology, transparent glass-ceramic (GC) with active nanocrystals is still required [1,2,3,4]. Specific optical properties of active GC materials resulting from two-phase structure (amorphous and crystalline) are found in the vicinity of lanthanide ions doped glassy host. The main benefits of a crystalline environment surrounding the rare earth ions are the high absorption and emission cross sections, lower phonon energy, and optimization of the ion–ion interaction [5]. The europium (Eu^3+^) ion is one of the interesting to analyze luminescence properties because: (i) it is characterized by relatively high quantum efficiency since the energy gap between the ^5^D_0_ emitting level and the underlying ^7^F_J_ multiplet is approx. 12,000 cm^−1^, which means that the non-radiative decay is less likely to occur even in hosts with high phonon energies such as phosphate and silicate glasses (1100–1200 cm^−1^); (ii) the intensity ratio between electrical-dipole (ED: ^5^D_0_→^7^F_2_) to magnetic-dipole (MD: ^5^D_0_→^7^F_1_) transitions can be used as a probe for site symmetry which is helpful to control ceramization process; and (iii) the Eu^3+^ ions-doped optical material can be used as an efficient phosphor for solid state light sources [6,7,8,9,10]. It is also well known that the intensities of emission bands of europium ion in glasses depend on its concentration and glass composition [11,12,13,14]. In order to obtain optimum emission characteristics for glass-ceramic applications, the influence of glass composition as well as concentration dependence studies of Eu^3+^ ions are essential. To date, a lot of photonic glasses after ceramization are presented in original papers [5,15,16,17,18,19,20]. Our idea is to modify thermally stable antimony-germanate glass with phosphorous oxide. Based on our earlier investigations on luminescent properties of antimony-germanate glasses doped with lanthanide ions we confirmed good thermal stability of glassy matrix. We also observed that the combination of two various glass-forming elements is particularly important in reduction of unfavorable non-radiative transitions between energy levels of rare-earth ions [21,22,23]. Thus, in case of glass-ceramic fabrication we decided to use P_2_O_5_ as a precursor of crystallization in antimony-germanate-silicate glasses doped with Eu_2_O_3_.

In the view of above, we demonstrated one-step method to obtain glass-ceramic material through the modification of antimony-germanate-silicate glass with a small amount of P_2_O_5_. The local symmetry in vicinity of Eu^3+^ ions and EuPO_4_ crystals creation were characterized by UV–vis spectrometry, X-ray diffraction (XRD), Scanning Electron Microscopy (SEM), and Energy Dispersive X-ray Spectrometry (EDS). It has been noticed, that the glass with 5 mol % of P_2_O_5_ shows band partition to emission sub-wavelengths of Eu^3+^ ions, which is characteristic effect in glass-ceramic materials. Also, the partial crystallization has been observed by naked eye directly after the melting process.

## 2. Results and Discussion

### 2.1. Analysis of Excitation and Emission Spectra

**Figure 1:**
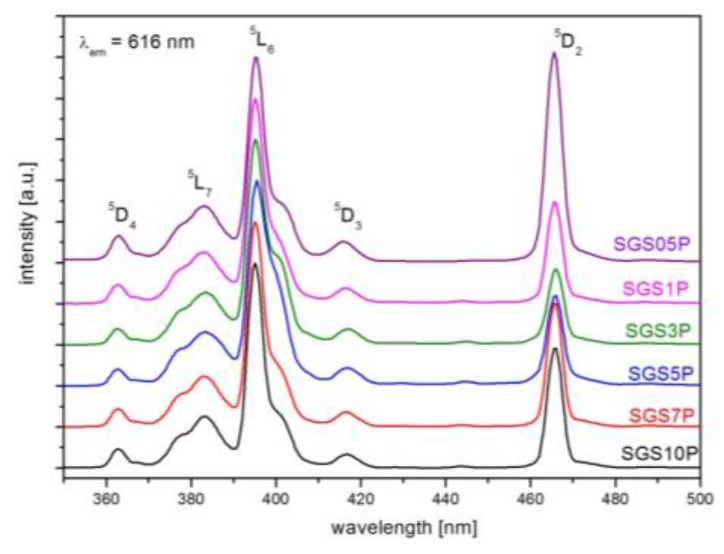
The excitation spectra of SGS05P, SGS1P, SGS3P, SGS5P, SGS7P, and SGS10P glass samples monitored at 616 nm.

Figure 1 shows excitation spectra of europium ions in the fabricated glasses, which have been monitored at the wavelength of 616 nm (^5^D_0_→^7^F_2_ transition). All glass samples are labeled due to phosphate oxide concentration as follows: SGS05P, SGS1P, SGS3P, SGS5P, SGS7P, and SGS10P. In analyzed spectral range (350–500 nm) five bands centered at the wavelengths 362 nm (^7^F_0_→^5^D_4_), 382 nm (^7^F_0_→^5^L_7_), 395 nm (^7^F_0_→^5^L_6_), 416 nm (^7^F_0_→^5^D_3_) and 464 nm (^7^F_0_→^5^D_2_) were observed. The band at 395 nm is characterized by most intense transition in UV–vis spectral range, hence it is suitable for the effective excitation of the Eu^3+^ ions by laser radiation at 394 nm. It worth to notice that intensity of second important excitation band at 464 nm in glass sample doped with 0.5 mol % P_2_O_5_ is more prominent. This band is called as the “hypersensitive transition” and strongly depends on structural changes of glassy matrix [24]. In our experiment, the increase of phosphate content in antimony-germanate glass leads to the strong reduction of “hypersensitive transition” intensity. Thus, the intensity change of ^7^F_0_→^5^D_2_ transition observed in fabricated glasses confirms the structural distortion of the glass network in the vicinity of Eu^3+^ ions.

**Figure 2:**
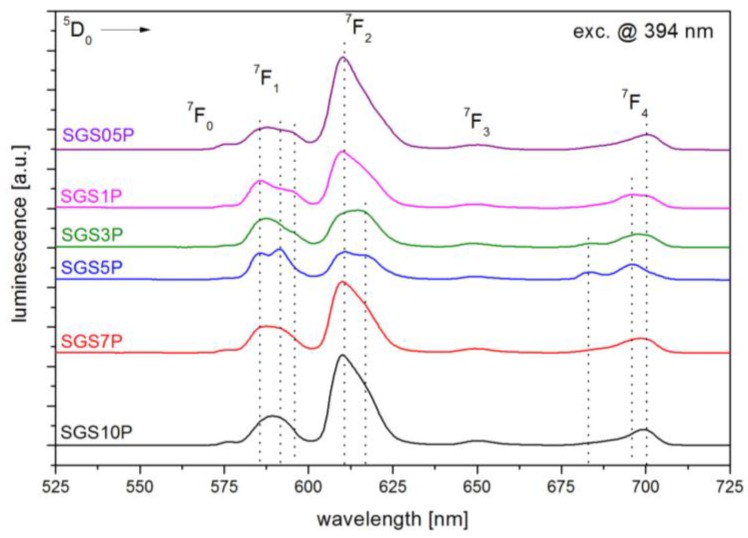
The luminescence spectra of SGSxP glasses doped with 0.5 mol % Eu_2_O_3_.

The luminescence spectra of fabricated antimony-germanate-silicate glasses doped with Eu^3+^ ions with different content of P_2_O_5_ under excitation by laser radiation with λ_exc_ = 394 nm were shown in Figure 2. In the range of 525–725 nm five characteristic emission bands have been observed at the wavelengths of 580, 594, 612, 653, and 703 nm originating from transitions of ^5^D_0_→^7^F_0_, ^7^F_1_,^7^F_2_, ^7^F_3_ and ^7^F_4_, respectively. All characteristics were scaled (integrated) to ^5^D_0_→^7^F_1_ transition due to their having independent features.

**Figure 3:**
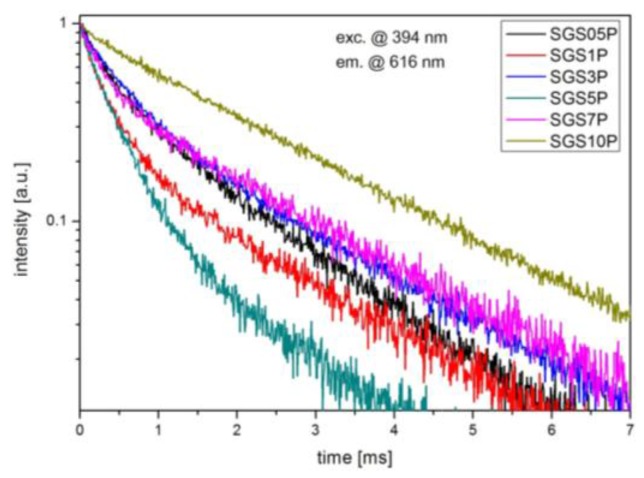
The luminescence decay of ^5^D_0_ energy level of europium ions in fabricated SGSxP glasses.

The changes in luminescence shape at 594 nm (^5^D_0_→^7^F_1_) and 616 nm (^5^D_0_→^7^F_2_) suggest that incorporation of P_2_O_5_ into glass matrix leads to the local structure modification of fabricated glasses. It worth to notice, that especially luminescence spectrum for SGS5P glass shows stark splitting for ^5^D_0_→^7^F_1_ and ^5^D_0_→^7^F_2_ transitions. Based on the Eu^3+^ ions luminescent properties and group-theoretical arguments it is possible to analyzed the structure of local environment in the vicinity of the ion [25,26]. In case of ^5^D_0_→^7^F_1_ transition three emission peaks resulting from the total removal of crystal field degeneracies were observed. The intensity of the ^5^D_0_→^7^F_2_ transition is higher than intensity of ^5^D_0_→^7^F_1_ transition in all samples except SGS5P, where dominant is transition ^5^D_0_→^7^F_1_. This result confirms that the local symmetry of Eu^3+^ ion is without an inversion center for SGS05P, SGS1P, SGS3P, SGS7P, and SGS10P samples and with an inversion center for SGS5P sample [27]. Additionally, the ^5^D_0_→^7^F_2_ transition is a “hypersensitive transition” which means that it strongly depends on the local symmetry and environmental effects in the surroundings of the Eu^3+^ ions [28]. Among the ^5^D_0_→^7^F_J_ transitions, the emission line at 594 nm (^5^D_0_→^7^F_1_) is a magnetic dipole transition (MD), while the emission line at 616 nm (^5^D_0_→^7^F_2_) is an electric dipole transition (ED). The ratio between integrated intensity of (^5^D_0_→^7^F_2_)/(^5^D_0_→^7^F_1_) transitions, gives a factor of the distortion degree from the inversion symmetry of local environment of the europium ions. This value is less than 1.0 for symmetric and is greater than 1.0 for non-centrosymmetric surroundings [14]. In our experiment, the ED/MD transition ratio decreases rapidly from 4.24 to 0.92 in function of P_2_O_5_ (up to 5 mol %), then starts to increase to approxximately 3 in glasses with 7 mol % and 10 mol % of P_2_O_5_ (Table 1). The relatively lower values of the ED/MD ratio for SGS5P suggest incorporation of Eu^3+^ ions into more symmetric environment with less covalent character [27]. Therefore, the ED/MD value is related to the enhancement in the symmetry of the ligand field around Eu^3+^ ions [29]. The variation in the asymmetric ratio and stark splitting for ^5^D_0_→^7^F_1_, ^5^D_0_→^7^F_2_ and ^5^D_0_→^7^F_4_ transitions in SGS5P sample may be due to the presence of the crystalline features [30]. Figure 3 presents the decay curves for all SGS glass samples excited at 394 nm, and the lifetime values obtained by fitting the curves with exponential functions are listed in Table 1. The luminescence decay curves of ^5^D_0_ energy level of Eu^3+^ in the glasses from SGS05P to SGS7P were fitted to the double-exponential fitting functions with a short decay (*τ_1_*) and a long decay (*τ_2_*). However, the luminescence decay curves of ^5^D_0_ state of Eu^3+^ in the SGS10P glass was best fitted to a single-exponential fitting function, which indicated a 1.99 ms lifetime. The luminescence intensity *I*(*t*) of the Eu^3+^ in glasses could be described by the sum of two exponential decay components from

(1) $$I\left(t\right)=A_{1}exp\left(-\frac{t}{\tau_{1}}\right)+A_{2}exp\left(-\frac{t}{\tau_{2}}\right)$$

where *τ*_1_ and *τ*_2_ were short- and long-decay components, respectively. Parameters *A*_1_ and *A*_2_ were fitting constants. According to Equation (1), the average lifetime <*τ_avg_*> was given by

(2) $$\langle \tau_{avg}\rangle =\frac{A_{1}\tau_{1}^{2}+A_{2}\tau_{2}^{2}}{A_{1}\tau_{1}+A_{2}\tau_{2}}$$

where weight factors *A*_1_ and *A*_2_ were introduced. According to Equation (2), the average lifetimes of ^5^D_0_ energy level of Eu^3+^ in the fabricated antimony-germanate glasses were calculated and presented in Table 1. It is well known that the luminescence of rare earth ions depended more or less efficiently on the molecular structure of the host. The luminescence decay rate was the sum of the radiative decay and multiphonon relaxation rates [31]. The non-radiative relaxation between various *J* states might occur by interaction of the electronic levels of rare earth ions with suitable vibrational modes of the environment [32]. Therefore, the double-exponential decay curves indicated that there were two different surroundings of the europium ions in the fabricated glass: some in the glass and the others in the nanocrystals. However, in the case of glasses with highest concentration of P_2_O_5_ (10 mol %) the luminescence decay has single-exponential character, which is a rather unexpected result and needs further structural investigations.

As the P_2_O_5_ concentration increases from 0.5 to 7 mol %, there is a slight increase of average ^5^D_0_ lifetime from 1.29 to 1.86 ms. In fact, the variation of lifetime is not monotonic and the shortest average lifetime of ^5^D_0_ level was obtained with the SGS5P glass sample. This effect could be related to the division of the excitation energy between Stark’s sub-levels. It is also well known in literature that the double-exponential decay indicates the energy transfer between Eu^3+^ ions by cross-relaxation process. Mainly in glasses with high concentration of Eu^3+^ ions. However, an interesting effect is that the SGS10P glass is characterized by longest lifetime (1.99 ms) than others glass samples. Based on these results, we confirmed that in fabricated glasses double-exponential decay only depends on concentration of P_2_O_5_.

### 2.2. X-ray Diffraction (XRD), Scaning Electron Microscopy (SEM), and Energy Dispersive X-ray Spectrometry (EDS)

**Figure 4:**
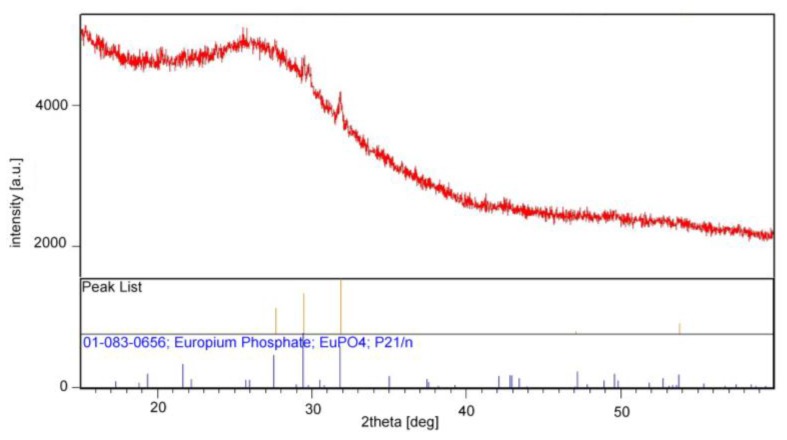
XRD patterns of SGS5P glass sample.

**Figure 5:**
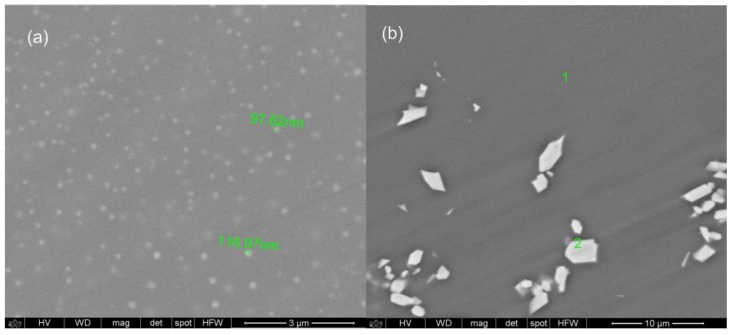
SEM image of SGS3P (**a**) and SGS5P (**b**) samples with approx. size of crystalline particles.

In order to confirm the presence of a crystalline phase or phases, the XRD measurements for SGS5P (lowest asymmetry ratio) sample was performed in the range from 15 to 60 degrees of diffraction angle (Figure 4). Broad halo effect between 2*Θ* = 20°–35° suggests the structural disorder and confirms dominance the amorphous nature of the SGS glass. However, in the diffraction pattern, three weak reflections peaks in the range of 2*Θ* from 28 to 31 degrees were also observed. After the reference analysis of peak positions calculated reflections are in good accordance with monoclinic EuPO_4_ crystalline phase (ICSD: 01-083-0656). From the peak width of the XRD pattern, the size of EuPO_4_ nanocrystals was estimated by the Scherrer formula

(3) $$D=\frac{\lambda K}{\beta cos\Theta }$$

where *D* is the crystal size at the vertical direction, *λ* is the wavelength of the X-ray, *Θ* is the angle of diffraction, *β* is the full width at half maximum (FWHM) of the diffraction peak and the instrument constant *K* = 0.9 [33]. The estimated average size of nanocrystals is about 30 nm. Figure 5 shows scanning electron microscope (SEM) images of SGS3P and SGS5P samples. As can be seen, in both SEM images there are visible crystals of a size range approx. 90–120 nm for the sample labeled as SGS3P (Figure 5a) and a few microns for the SGS5P sample (Figure 5b).

In the case of the SGS5P sample, the analysis of the chemical composition in two different areas (EDS for amorphous—1; and for crystalline—2) shows that the microparticles observed in the SEM image originate from crystals of europium phosphate (supplementary file—Figure S1).

In the case of SGS3P sample, due to the nanometric size of the particles is not possible to obtain an EDS spectrum separately for crystalline and the amorphous area. However, due to very similar chemical composition of the analyzed glass samples, it can be assumed that EuPO_4_ crystals have been also observed.

## 3. Materials and Methods

### 3.1. Glass Preparation

Antimony-germanate-silicate (SGS) glasses have been investigated in our earlier works due to good thermal stability, which is required in the optical fiber fabrication process [22,23]. Here, we proposed the modification of SGS glass by small amount of P_2_O_5_ from 0.5 mol % to 10 mol %. The glass with molar composition 25Sb_2_O_3_–25Ge_2_O_3_–10Al_2_O_3_–5Na_2_O–(35-x)SiO_2_–xP_2_O_5_–0.5Eu_2_O_3_ was synthesized by standard melt-quenching method. The Eu_2_O_3_ concentration was fixed on the level of 0.5mol % and glasses are labeled as: SGS05P, SGS1P, SGS3P, SGS5P, SGS7P, and SGS10P. All the raw materials were analytical grade reagents (99.99%). Europium ions were used as a local environment probe, to obtain valuable information on the site symmetry of active ions in a fabricated host directly by luminescent measurements. A homogenized set was placed in a platinum crucible and melted in an electric furnace at 1450 °C for 60 min in oxygen atmosphere. Next, the glass melt was poured into a brass plate at the room temperature (RT) and then annealed at 400 °C for 12 h to release the internal stress from the quench. Next, glasses were cooled down to room temperature and polished carefully in order to meet the requirements for optical measurements. After this, in a few glass samples, the partial-crystallization (opaque) phenomenon and agglomeration of crystals were observed by naked eye.

### 3.2. Structural and Spectroscopic Analysis

The XRD patterns of fabricated glasses were measured in the range from 10° to 80° using an X'Pert Pro diffractometer (PANalytical, Eindhoven, Netherlands). The Cu X-ray tube with K_α_ radiation was used. The morphology of prepared samples was examined by FEI Company (Hillsboro, OR, USA) Nova Nano SEM 200 scanning electron microscope with an attachment for chemical analysis with energy dispersive X-ray spectroscopy (EDX, EDAX). The analyses were carried out in secondary electron mode (SE). Prior to analyses, the samples were covered with carbon layer. The excitation and luminescence spectra of the glasses in a range of 350–750 nm were measured using a JobinYvon Fluoromax4 spectrophotometer (Horiba, Kyoto, Japan). A system PTI QuantaMaster QM40 coupled with tunable pulsed optical parametric oscillator (OPO), pumped by a third harmonic of a Nd:YAG laser (OpotekOpolette 355 LD, Carlsbad, CA, USA) was used for luminescence decay measurements. The laser system was equipped with a double 200 mm monochromator, a multimode UV–vis PMT (R928) (Hamamatsu, Japan) and H10330B-75 detectors (Hamamatsu, Japan) controlled by a computer. Luminescence decay curves were recorded and stored by a PTI ASOC-10 (USB-2500) oscilloscope with an accuracy of ±1 µs.

## 4. Conclusions

As a result of our experiment we synthesized the antimony-germanate-silicate glass modified by different content of P_2_O_5_. Incorporation of phosphate oxide into host enables to create monoclinic crystal phase EuPO_4_ directly in melt-quenching process (without additional heat-treatment). Also, the effect of partially crystallization leads to prominent Stark splitting of luminescence bands at 594 nm and 616 nm, which suggests that Eu^3+^ ions are surrounded by crystalline phase. According to SEM/EDS measurements, the formation of europium phosphate crystals with nano- and micrometric size have been also observed.

## Figures and Tables

**Table 1:** The ED/MD transition ratio, lifetimes (*τ*_1_ and *τ*_2_) and average lifetime <*τ_avg_*> of ^5^D_0_ level of Eu^3+^ ions.

Glass Sample	ED/MD Ratio	<*τ_avg_*> [ms]
SGS05P	4.24	1.29
SGS1P	2.02	1.29
SGS3P	1.27	1.86
SGS5P	0.92	0.83
SGS7P	2.66	1.72
SGS10P	3.03	1.99

## Supplementary Materials

The following are available online at http://www.mdpi.com/1996-1944/10/9/1059/s1, Figure S1: EDS spectra of SGS5P glass for amorphous (1) and crystalline (2) areas.
