# Supplementary figures and images for: Optical Characterization of Nano- and Microcrystals of EuPO4 Created by One-Step Synthesis of Antimony-Germanate-Silicate Glass Modified by P2O5
Source: Materials (Basel). 2017 Sep 9;10(9):1059. doi: 10.3390/ma10091059 (PMC5615714; doi:10.3390/ma10091059)

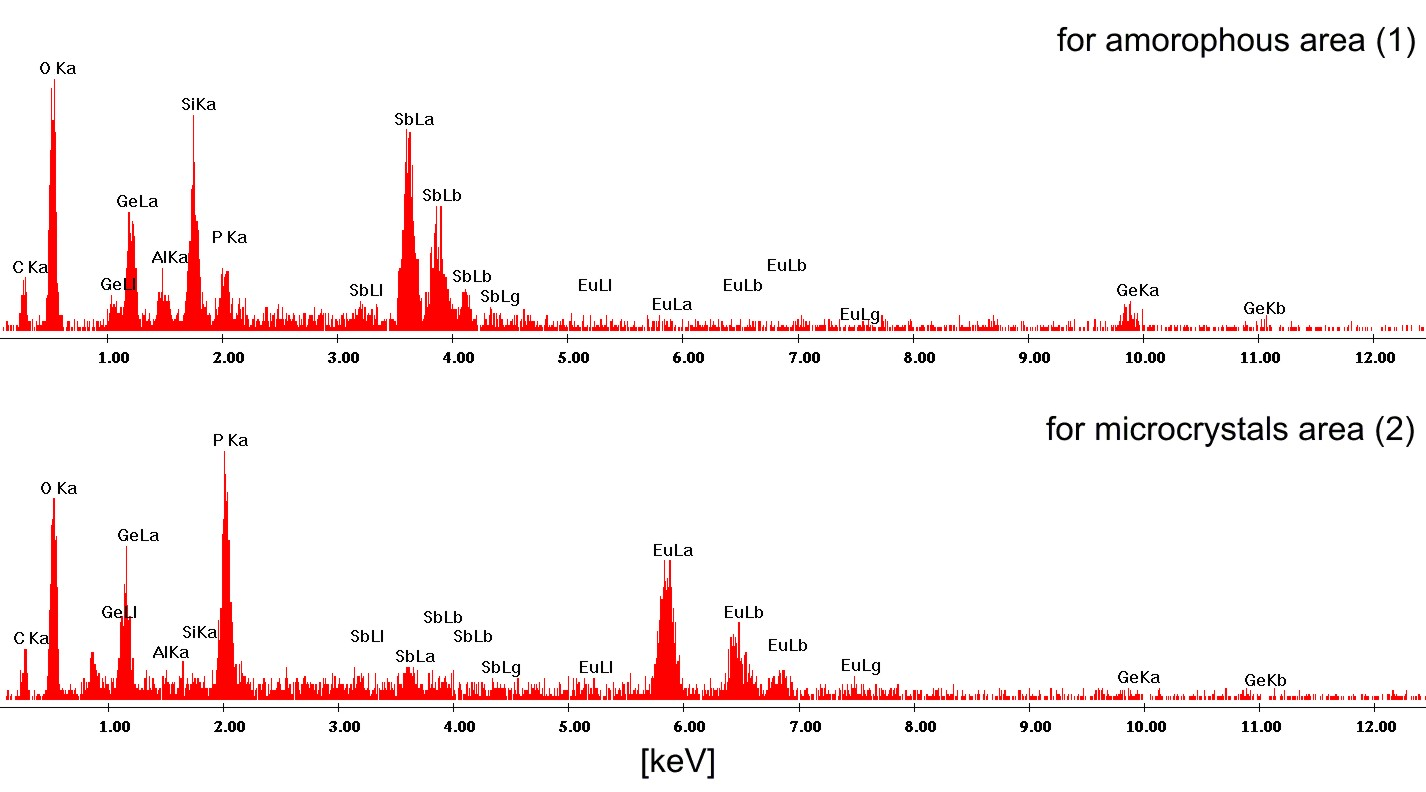

Supplement: Supplementary file 1 [file materials-10-01059-s001.zip › Fig_S1.tif]
